# Prone Positioning and Intravenous Zanamivir may Represent Effective Alternatives for Patients with Severe ARDS Virus A (H1N1) Related Pneumonia in Hospitals with no Access to ECMO

**DOI:** 10.1155/2010/146456

**Published:** 2010-12-15

**Authors:** Giuseppe Gristina, Giuseppe Nardi, Daniela Orazi, Francesco Nicola Lauria, Maria Beatrice Valli, Eleonora Lalle, Stefano Menzo, Luigi Riccioni, Maria Pia Camporiondo

**Affiliations:** ^1^Intensive Care Unit 1 Shock and Trauma Department, Azienda Ospedaliera San Camillo-Forlanini, Viale Gianicolense 87, 00152 Roma, Italy; ^2^Department of Hygiene and Infection Control, Azienda Ospedaliera San Camillo-Forlanini, Viale Gianicolense 87, 00152 Roma, Italy; ^3^Clinical Departement, Istituto Nazionale per le Malattie Infettive, Lazzaro Spallanzani-Roma, Via Portuense, 00149 Roma, Italy; ^4^Laboratory of Virology, Istituto Nazionale per le Malattie Infettive, Lazzaro Spallanzani-Roma, Via Portuense, 00149 Roma, Italy; ^5^Department of Microbiology and Molecular Biology, Azienda Ospedaliera San Camillo-Forlanini, Viale Gianicolense 87, 00152 Roma, Italy

## Abstract

The first patient with influenza A/H1N1-related pneumonia was admitted to an Italian ICU at the end of August 2009. Until then, despite the international alarm, the level of awareness was low and very few Italian hospitals were equipped with ECMOs. Moreover the PCR test for A H1N1 virus was sporadically available and the emergency departments of even the largest institutions could rely only on the rapid test for the urgent screening of patients with pneumonia and respiratory failure. On September 5th, a young and “apparently” previously healthy man, was admitted to our ICU because of a severe ARDS caused by influenza A H1N1 virus. As there was no ECMO available, he was treated with prolonged cycles of prone positioning ventilation. Antiviral treatment was started with Oseltamivir, but as enteral absorption was impaired by paralytic ileus and tube feeding intolerance, Oseltamivir had to be discontinued. Intravenous Zanamivir 1200 mg/day for ten days was therefore prescribed as “off label” antiviral therapy. A bone marrow biopsy allowed the diagnosis of an initial stage of “hairy cells leukaemia.” ARDS related to A/H1N1 influenza was the first sign of the disease in our patient. He did well with complete clearance of the infection from the BAL after 10 days of Zanamivir, although the nasopharyngeal swabs remained positive for ten more days. Prone positioning ventilation may be a life-saver strategy in patients with severe ARDS when ECMO is not immediately available. However, prone positioning ventilation is often associated with severe impairment of the absorption of drugs that require enteral administration via the nasogastric tube. In these cases, intravenous Zanamivir may be an effective alternative strategy.

## 1. Case Report

On September 5th, a 41-year-old Caucasian man was transferred to our Emergency Department (ED) from a nearby private hospital, where he had been admitted only a few hours before. 

There was nothing relevant in his past medical history. He was under no medication and had no history of alcohol or drug abuse. His body weight and height were, respectively, 90 Kg and 190 cm. 

Three days earlier, he called the outpatient department of a district hospital complaining of high fever (40°C), cough, and myalgia. In spite of a chest X-ray showing bilateral infiltrates, he was sent home on antibiotics with no further investigations prescribed. 

In the next three days, he worsened progressively. On admission to our ED, he was dyspnoeic and hypoxic with respiratory fatigue, tachycardia, and systolic hypertension. 

Blood gas analysis on admission showed severe hypoxemia, hypercarbia, and respiratory acidosis.

He was given high-flow oxygen; blood cultures were performed as well as a pharyngeal swab for an H1N1 rapid test. The urines were tested for Streptococcus Pneumoniae and Legionella free antigens. 

The chest CT scan showed “ground glass” bilateral infiltrates involving the dependent regions of both lungs, suggesting ARDS pneumonia related. 

After sedation, the patient's trachea was intubated. A tracheal aspirate was sent for Gram staining and culture. Antibiotic therapy was modified and different drugs were prescribed according to the local guidelines for “nonresponding pneumonia” (Piperacillin/Tazobactam + Vancomycin + Levofloxacin).

Bacterial urinary antigens were negative as well as the Gram stain on the tracheal aspirate. The A virus H1N1 rapid test (Binax-Now, Inverness Medical, Southgate, Maine, USA) was performed on admission to the ED and repeated 12 hours later. Both tests were negative. The patient was admitted to our ICU. 

On admission to the ICU, the patient was sedated and artificially ventilated. Blood Pressure (90/50) was supported with vasopressors (Norepinephrine 9 mcg/Kg/min) and hynotropic agents (Dobutamine 28 mcg/Kg/min). Urinary output was preserved. Body temperature was 39°C. On peripheral blood sample, leukopenia (<700) was recorded.

A Pressure Control Ventilation-Volume Guaranteed (PCV-VG) with protective lung strategy (8 ml/Kg) was started.

The initial P/F was as low as 84, and increasing oxygen concentrations were required. 

Positive End Expiratory Pressure (PEEP) was set up to 16 cm H_2_O (best PEEP) with only slight improvement. In the next few hours, haemodynamic monitoring with PiCCO was started, and Trans-Thoracic Echocardiography (TTE) was performed.

TTE showed preserved global systolic function, high right ventricular telesystolic pressure despite right ventricle normal thickening with paradoxical septal motion, and limited tricuspid regurgitation. 

These results were consistent with the data of PiCCO (low cardiac index, high total peripheral resistances, high lung water, low preload volume, and high stroke volume variation). 

During the patient ICU stay, serum creatinine was in a normal range (0.5–1.5 mg/dL) demonstrating a normal renal function. 

Sedation was obtained using midazolam 5 mg/h plus fentanyl 0.15 mg/h continuously to achieve a Ramsay score 3-4. 

A bone marrow biopsy was performed to investigate the severe leukopenia as well as a new BAL. BAL samples were sent for culture and for nucleic acid testing for Legionella, Chlamydia, Cytomegalovirus, influenza virus, and Pneumocystis Carinii, together with nasopharyngeal swabs for influenza RT-PCR.

**Figure 1:**
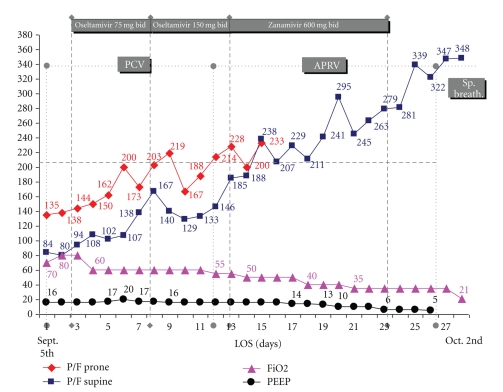
Correlation among P/F values in supine and prone positions, PEEP levels, and ventilation modalities.

Because of further worsening in hypoxemia, prone positioning was started after inducing more deep sedation and muscular paralysis using Vecuronium Bromidii 2 mg/h continuously. The prone position was associated with a significant improvement in the gas exchange as well as with a sharp amelioration in the patient's haemodynamics (Figure 1). At the same time, enteral nutrition (1 Kcal/mL–1000 mL/q.d.) and hydration (1000 mL/q.d.) were used as a conservative strategy fluids intake; antithrombotic therapy (LMWH 4000 U sc/q.d.), methylprednisolone (1.0 mg/kg 6 i.d.) were started. Each time the patient was turned back to the supine position (as we planned to do every 12 hours), a fast deterioration in the P/F was observed together with worsening of the cardiac output and of the patient's haemodynamics. Therefore, prolonged cycles of prone positioning ventilation were alternated with short intervals in supine. On day 3, influenza virus A(H1N1)v was identified by a qualitative RT-PCR (CDC protocol of real-time RT-PCR for influenza A(H1N1), WHO April 2009) in nasopharyngeal swabs. Oseltamivir 75 mg bid was started via nasogastric tube (NGT) accordingly with the suggested dose from pharmaceutical company. 

The patient showed a progressive haemodynamic improvement, and while kept in the prone position he could be weaned from hynotropic agents and vasopressors. In the supine position, Cardiac Output still required dobutamine support. 

However, prone position maintenance needed deep sedation and muscular paralysis for most of the time. In our opinion, gastric distension refractory to prokinetics, vasopressors and GI edema due to SIRS, and tube feeding intolerance made the administration of Oseltamivir via nasogastric tube unreliable rather than prone position itself. 

The bone marrow biopsy showed an early stage of “hairy cells” leukaemia. There were no signs or symptoms of the disease in the recent patient's medical history. A routine blood analysis only a few months before was completely normal. No specific therapy was prescribed but lenograstim (Granulocyte). WBC count raised up from 350/mm^3^ to 4000/mm^3^ within 5 days. On day 11 and 12, he could be kept supine 24 hours a day. 

On day 8, the temperature increased and the respiratory patterns worsened. A new qualitative RT-PCR turned out positive for virus A(H1N1)v. The dosage of Oseltamivir was doubled (150 mg × 2). Prone positioning had to be started again, and a PEEP up to 20 cm H_2_O was required. Once again feeding intolerance and vomiting hampered the nasogastric administration of Oseltamivir. 

On day 11, another qualitative pharyngeal swab showed a persistence of the influenza virus A(H1N1)v. Resistance testing was performed on day 12 by direct sequencing of a significant portion of the whole gene: no single mutation was detected compared to the sequence from the day 2 sample, either at critical sites or anywhere in the sequence. As the patient still needed to be ventilated in prone position, we decided to discontinue oral Oseltamivir. The permission to use Zanamivir via the intravenous route to avoid gastric intolerance was requested to the patient's relatives and the Hospital Ethical Committee. The “off label” use of Zanamivir 600 mg IV bid for 10 days was authorized, and the drug was started on day 13.

During the following days the patient slightly improved. All bacteriological investigations (blood, urines, and tracheo-aspirates) were negative throughout the ICU stay with the exception of a single isolate of candida albicans from a central venous line. Percutaneous tracheostomy was performed on day 16, as soon as patient's oxygenation improved enough to allow this procedure to be safely performed. On day 13, the ventilation was shifted from Pressure Controlled to Airway Pressure Released Ventilation (APRV) and subsequently to Pressure Support with a progressive decrease in the levels of PEEP and FiO_2_. Once sedation was discontinued, effective spontaneous ventilation (Sp. Breath.) was restored with good recovery of autonomous respiratory function. Figure 1 shows the trend in P/F and duration of any ventilation setting we used.

Since influenza virus persisted in respiratory swabs, a new resistance test was performed on day 18, but this time analyzing most of the neuraminidase gene sequence again without any mutation detected.

On day 22, a bronchoalveolar lavage (BAL) sent for virus A(H1N1) was negative. Zanamivir was discontinued. The tracheostomy was closed, and the patient was transferred to the ward. Eight days later, on October 10th, he was sent home and subsequently followed as an outpatient.

**Figure 2:**
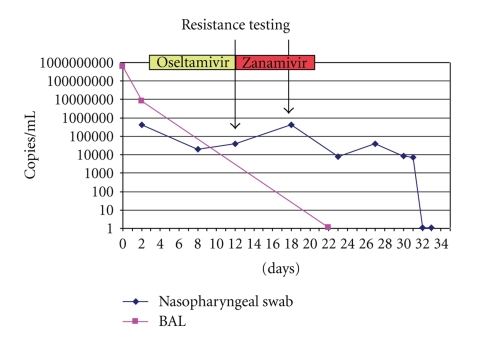
Modifications of the viral charge during treatment with oral Oseltamivir and parenteral Zanamivir.

During the patient ICU stay, all the BAL and nose-throat samples were analyzed by a qualitative real-time PCR. Subsequently, the amplification curves were quantitatively reevaluated with the aid of an external calibration curve standardized on the positive control (a series of aliquots of the same material from an *in vitro* culture, used in all sessions), in order to determine the viral load dynamics in respiratory samples and how it was influenced by the treatment.  Figure 2 shows the modification in the viral load during the ICU stay.

## 2. Discussion

Death from influenza A(H1N1)v pneumonia is related to the onset of ALI-ARDS [1, 2].

Although our young patient had a negative medical history, he had an underlying lymphoproliferative disease which may explain why pneumonia rapidly worsened evolving to such a severe ARDS [3–6].

In our experience, the use of Oseltamivir through the nasogastric route was associated to a 20-fold reduction of viral load in nasopharyngeal secretions, as shown in Figure 2. It is interesting to note that in our case Oseltamivir was started on day 3 because of a false negative result derived from a quick virus test. However, the role of the drug could not be demonstrated as the viral load could unfortunately only be evaluated “a posteriori”. For this reason, we interpreted the persistence of the positive tests on days 8 and 11 as the consequence of suboptimal absorption of Oseltamivir. Indeed, the inflammatory response affecting also the gastrointestinal mucosa and the combination effect of deep sedation/paralysis and prone position severely delayed peristalsis (high-volume gastric aspirates), possibly impairing drug absorption. For these reasons, we decided to treat the patient with parenteral Zanamivir.

In our experience, intravenous Zanamivir (aqueous solution) was safe [7–9]. The administration of Zanamivir (1200 mg/day for ten days) was associated with a progressive clinical recovery followed by the complete eradication of the virus from the BAL. A similar result was observed by Kidd et al. [9]. However, Zanamivir was unable to clear or reduce viral load from nasopharyngeal secretions during treatment. It is therefore questionable whether viral clearance in BAL was a simple chronological association or the genuine antiviral effect of intravenous Zanamivir. As the “off label” therapy with Zanamivir was only authorized for ten days, the drug was discontinued before complete viral clearance was achieved. 

Zanamivir in aqueous solution is still unlicensed. This leads to difficulties in the drug supply and to the need of specific authorization before its administration: we used Zanamivir in agreement and under the supervision of the Hospital Ethical Committee. 

As we know, there is a mounting evidence that ECMO may improve survival in patients suffering ARDS from H1N1 influenza [10]. 

Before the onset of the influenza A(H1N1)v pandemic, the use of ECMO in Italy was limited to a few institutions, and even many tertiary care hospitals were not equipped with ECMO. Other European countries may face a similar situation as not a single patient with A H1N1 virus-related respiratory failure was treated with ECMO in Spain according to the recent paper from Rello et al. [11]. An ongoing Italian national program will probably bring a fast diffusion of ECMOs as a tool to treat patients with ARDS caused by A(H1N1)v.

Once a patient presents with a very severe ARDS, there may be major problems to refer him to other hospitals as prolonged transportation may be extremely dangerous. In these cases, an experienced team may effectively provide an adequate respiratory support by means of a protective lung strategy and a wise use of the PEEP. In our experience, prolonged cycles of prone positioning ventilation have been the key to ensure a proper oxygenation and ameliorate cardiac output. 

Our patient was submitted to prolonged cycles (at least 12 h hours each) of prone positioning ventilation for as long as 15 days. Very often the interval between two cycles of prone positioning could not be longer than two hours to avoid gas exchange deterioration and the worsening of patient's haemodynamics. The PEEP was continuously adjusted looking for the best PEEP. Tidal volume was strictly maintained within the 6–8 ml/kg range. Airway pressure release ventilation was started as soon as possible to reduce the risk of baro/volutrauma. Methylprednisolone was administered since day 2 and throughout the ICU stay. 

There is no consensus in the literature as for the use and dosage of steroids in virus A H1N1 pneumonia and steroids are not included in the treatment guidelines [12]. 

However, a few controlled trials and at least one meta-analysis support the use of corticosteroids in ARDS [13]. Steroids used early and in doses not exceeding 1 mg/kg/day may improve mortality and reduce ICU length of stay. According to Meduri et al. [13], steroids may even decrease bacterial superinfections through a reduction of lung inflammation.

In addition, the best critical care standards (haemodynamics, diuresis, fluid balance, sedation, nutrition, and infections prevention) have a dramatic relevance to avoid further complications and prevent mortality.

Although there are no definitive recommendations for the treatment of patients with severe lung injury related to virus A/H1N1 infection, these patients should be probably managed not differently from patients with ARDS due to other causes. The antiviral drugs are recommended to reduce viral concentration. There are no data about the effectiveness of the different antiviral agents. Drug absorption may be important to ensure effective blood levels of the administered antiviral drug. In our experience, feeding intolerance might impair enteral absorption, intravenous Zanamivir was an effective substitute for oral Oseltamivir.

## 3. Conclusions

Prone positioning ventilation may be a life-saver strategy in patients with severe ARDS when ECMO is not immediately available. However, prone positioning ventilation is often associated with severe impairment of the absorption of drugs that require enteral administration via the nasogastric tube. In these cases, intravenous Zanamivir may be an effective alternative.
